# Development of a polymeric biomedical device platform with controlled disassembly and in vivo testing in a swine intestinal model

**DOI:** 10.1038/s41598-022-06339-9

**Published:** 2022-02-25

**Authors:** Karrer M. Alghazali, Alisha P. Pedersen, Rabab N. Hamzah, Pierre-Yves Mulon, Rebecca E. Rifkin, Anwer Mhannawee, Zeid A. Nima Alsudani, Christopher Griffin, Malek A. H. Muhi, Nikki Mullen, Robert L. Donnell, David E. Anderson, Alexandru S. Biris

**Affiliations:** 1 Center for Integrative Nanotechnology Sciences, University of Arkansas at Little Rock, Little Rock, AR USA; 2 NuShores Biosciences LLC, Little Rock, AR USA; 3 Department of Large Animal Clinical Sciences, College of Veterinary Medicine, University of Tennessee, Knoxville, TN USA; 4 Department of Biomedical and Diagnostic Sciences, College of Veterinary Medicine, University of Tennessee, Knoxville, TN USA

**Keywords:** Biomaterials, Materials for devices

## Abstract

The aim of this study was to create a surgical guide platform that maintains its integrity while the surgeon performs an intestinal anastomosis or another similar procedure, which then breaks apart and is eliminated from the body in a controlled manner. The device contains mixed polymeric structures that give it a controlled rate of disassembly that could meet the requirements of a specific surgical purpose. The intraluminal anastomotic guide was manufactured as a hollow cylinder composed of layers of porous polyurethane/PCL with polyvinylpyrrolidone as the binding agent similar to a “brick–mortar” architecture. This combination of polymeric structures is a promising manufacturing method from which a variety of tunable devices can be fabricated for specific medical procedures and site-specific indications. The guide was designed to rapidly disassemble within the intestinal lumen after use, reliably degrading while maintaining sufficient mechanical rigidity and stability to support manipulation during complex surgical procedures. The nature of the device’s disassembly makes it suitable for use in hollow structures that discharge their contents, resulting in their elimination from the body. A swine model of intestinal anastomosis was utilized to validate the use and function of the device.

## Introduction

Biomedical devices are typically designed to either remain in situ permanently or degrade relatively slowly over months to years^1^. However, in some situations, a medical implant is needed for only short periods of time—hours to days—to aid in a surgical procedure. In these cases, long-term retention of the device within the body is not desired and may be detrimental. Complications may include infections, foreign body reactions, or other morbidities. Thus, medical devices that only need to be retained for short periods of time are best suited to tissues that discharge their contents outside of the body, such as the esophagus, intestinal tract, pancreatic duct, bile duct, lactiferous ducts, tear ducts, and nasal cavity. Recently, we reported the successful creation and testing of a novel polymeric platform technology that features precision-tuning of controlled collapse characteristics. The first platform matured into a clinical device designed for use in end-to-end intestinal anastomoses and is referred to as an anastomotic guide (AG). The function of the AG is to improve the surgical technique and enhance the surgical support and procedural ease of hand-sewn anastomosis of the small intestine^2–9^.

An intraluminal guide for intestinal anastomosis was chosen to be the platform’s first-generation device because intestinal anastomosis is a common procedure generally associated with significant morbidity rates^2–8^. Performed in both human and veterinary patients, intestinal resection and anastomosis have a variety of indications, including obstruction, such as from foreign bodies, pathologic strictures, or chronic constipation; inadequate segmental functionality due to neurologic dysfunction; traumatic or ulcerative perforation; or other disease processes, including intussusception, neoplasia, volvulus, torsion, chronic inflammatory bowel disease, and Crohn’s disease^2,3^. Intestinal anastomosis often includes resection of a diseased segment of bowel prior to reconnection of the remaining viable ends in order to reestablish bowel continuity^2,3^. Factors that affect the healing and ultimate outcome of an intestinal anastomosis include local blood perfusion, apposition and alignment of the cut edges of bowel, tension at the anastomotic site, presence of contamination, and, most importantly, surgical technique used^4,8^.

Despite a variety of minimally invasive and stapled anastomosis techniques available, hand-sewn end-to-end anastomosis of the small intestine remains a common technique for re-establishing intestinal patency and flow of digesta after intestinal resection^10–12^. Post-operative complications are commonly encountered, the most significant being dehiscence, leakage, peritonitis, ileus, tissue necrosis, obstruction, tissue hypoxia, stricture, and death^2–6^. Dehiscence and leakage from the anastomotic site, often causing septic peritonitis, is one of the most serious complications and is reported to occur in as many as 1 to 24% of cases^7,8^. Additionally, stricture of the intestine at the anastomotic site is a routine consequence of intestinal resection and anastomosis, regardless of the technique used^6^. Non-degradable metal or plastic intraluminal stents have been utilized to address strictures, but there are numerous associated morbidities, including secondary stricture, stent migration, hyperplasia of intestinal mucosa, perforation by the stent through the intestinal wall, and the necessity for repeated endoscopic procedures^6^. Despite medical advancements, including automated devices, these complications persist^12^.

The ideal technique for intestinal anastomosis would achieve primary healing with the cut edges of bowel in precise apposition to one another, maintain local vasculature, eliminate foreign material at the surgical site, and apply sufficient tensile force when placing sutures to keep tissues aligned without gap or dehiscence^4,5,9,11,13^. When intraluminal support is only needed during the immediate operative period and not continually during the convalescent period, a rapidly degradable intraluminal medical device would be vital to minimize complications of hand-sewn anastomosis and prevent procedural- and device-associated morbidities. Such a device is expected to greatly reduce morbidities that require placement of an indwelling non-degradable stent at a later date.

Complex biomaterials and bioprocesses are increasingly being investigated as alternatives for traditional indwelling medical devices because of the ability to control their properties and characteristics, as well as the frequency of morbidities associated with devices that remain in the body long-term^6,14^. Benefits of utilizing biomaterials for an AG include potential for rapid degradation, lack of interference with intestinal motility, and elimination of dislodgement and obstruction concerns. Some biodegradable stents have been proposed and fabricated, but the typical timeframe that these remain in the intestine (weeks to months) exceeds that which is needed for the anastomotic procedure and may present morbidities of their own^6^. For example, intraluminal stents composed of magnesium alloys have a high corrosion rate^6^. Polymer-based stents, such as those composed of poly(l-lactide), polydioxanone (PDS), or glycolide-co-ε-caprolactone, are reported to have degradation rates ranging from weeks to months and are at risk of dislodgement during this timeframe^6^. Several case series utilizing PDS-based stents revealed migration rates ranging from 0 to 36%^6^. Kuo et al.^13^ assessed the use of a short-duration agarose-based stent for intestinal anastomosis in rabbits. Anastomoses performed with the agarose stent resulted in significantly shorter operating times, greater collagen deposition and vessel formation at the anastomotic site, and increased bursting pressure of the anastomosis 21 days after surgery, compared with anastomoses performed without the stent. This supports the potential benefits of using an intraluminal guide for the procedure without the need for sustained presence of stents.

Our first-generation prototype AG was created by a multi-layer approach and successfully tested in a swine model of intestinal anastomosis^9^. We showed that the AG aided the performance of the procedure and then rapidly softened, collapsed, and was passed out in feces, as designed. This platform’s specifications may allow it to be utilized in a variety of tissues, indications, and surgical procedures. We propose several benefits to incorporating a rapidly disassembling AG into the hand-sewn technique. First, the AG is designed to be used with the same hand-sewn suturing technique that surgeons are accustomed to, making the procedure easier and less prone to complications. The AG expands the lumen of the bowel so that the edges that frequently evert after transection resume a more normal conformation. When suturing results in an inverted or everted anastomosis, the size of the intestinal lumen is reduced. Additionally, mucosal eversion may increase the incidence of adhesion development^2^. Visualization of the delineation between the layers of the bowel wall is enhanced with the AG, ensuring that submucosa is contained in each suture. This is imperative, as the submucosa is the holding layer of the intestinal anastomosis and is the most resistant to the tensile forces exerted on the site^4,5^. The AG’s expansion of the bowel allows the entirety of the anastomotic site to be better visualized and ensures that no aspect of the circumference is missed in the anastomosis, including at the mesenteric border, where most post-operative anastomotic leaks occur^5^. This enhanced visualization is important not only for assurance of complete circumferential closure of the anastomosis but also for reducing excessive suture material that may be incorporated due to concerns about tissue integrity^4^. The guide eliminates the possibility of engaging the posterior wall of the intestine during placement of sutures, a recognized complication of hand-sewn anastomosis when the bowel is collapsed during the procedure^5^. In addition, the dimensions of the intestinal lumen allow for ease of device design and fabrication, and the movement of digesta allows for use of a device intended for short-term utility.

Based on the positive results of our first-generation AG device and the clinical need for a rapidly degradable AG, we sought to improve our device’s functionality by refining the architecture and the rate of disassembly into small segments. The objective was to create an anastomotic guide that would disassemble within hours after hydration and implantation. For this next-generation AG, we chose polymer-based structures that would contribute to rapid degradability and have demonstrated biocompatibility; we also utilized newly developed integrative manufacturing processes that would allow us to reliably obtain such degradable AGs. The devices were tested ex vivo and in a well-established swine model of small intestinal anastomosis in order to prove their in vivo functionality and assess usability, handleability, and ability to disassemble upon hydration and be eliminated from the body. We hypothesized that the AG would disassemble during the immediate post-operative period and be passed out in feces without disturbing intestinal recovery. Further, we expected that the AG would enhance the technical execution of the surgical procedure. Our results clearly indicate that the newly developed AG does degrade in the desired timeframe and that all its components are eliminated from the animals without any complications. Furthermore, the AGs offered good handleability during the surgical procedures.

## Materials and methods

### Anastomotic guide fabrication and hydration/degradation testing

#### Anastomotic guide fabrication

**Figure 1:**
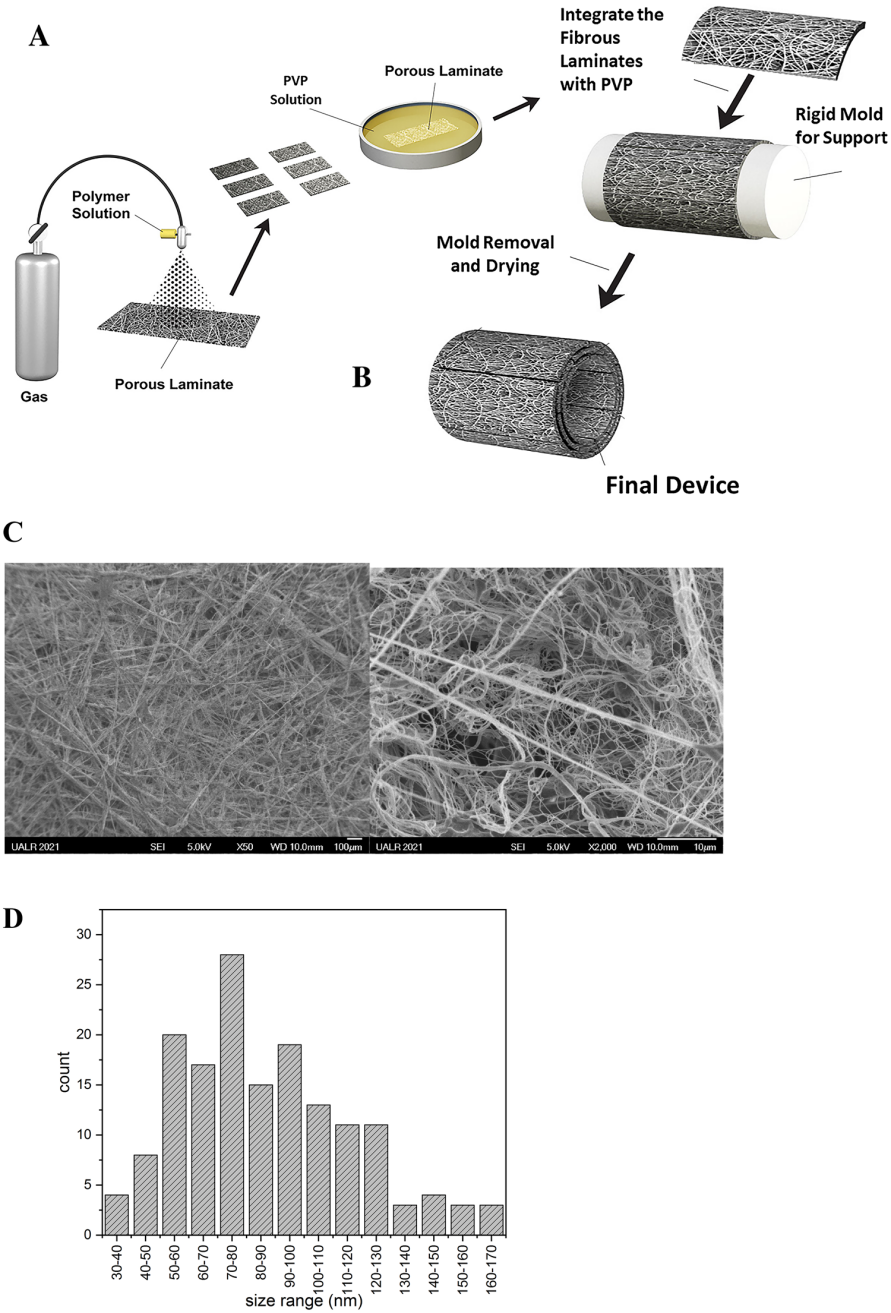
Device fabrication steps: (**A**) the porous polymer laminates were saturated with adhesive solution then assembled over support mold; (**B**) the support mold was removed and the device left to dry for 48 h; (**C**) low (left) and high (right) magnification SEM images of the polymer films used for the formation of the porous laminates; (**D**) diameter size histogram for the as-sprayed fibers, as measured with ImageJ software with a total of n = 159 entry points.

**Figure 2:**
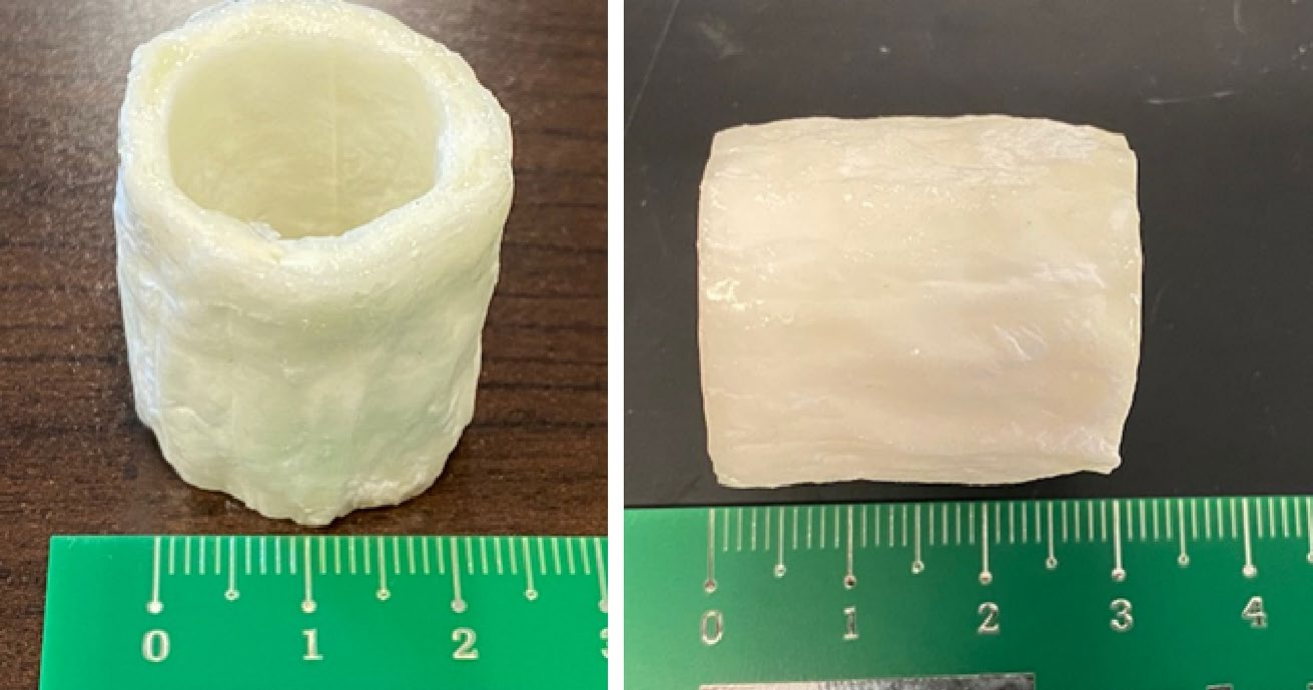
(**A**) pre-sterilized AG viewed obliquely; (**B**) pre-sterilized AG viewed end-on. It can be observed that the non-smooth and corrugated surface was designed to assist the surgeon during the surgery by limiting the slipping of the soft tissue over its surface.

The device (patent pending, application number PCT/US2019/041550) was fabricated by assembling layers of porous polymer laminate to the form of a cylindrical shape. We have developed a novel fabrication and design approach that involves the multistructural assembly of highly porous, water-absorbing polymer sheets built into a cylindrical shape and held in place by a second polymer that has fast water solubility (Fig. 1). Basically, we employed the “brick and mortar” construction analogy. The films produced by controlled air spraying were cut into laminates of rectangular shapes and uniform dimensions that were chosen such that they would result in the fabrication of devices of desired lengths. Briefly, a novel method was used to fabricate the porous polymer film. The technique uses high-velocity spraying to form a microfiber structure of the polymer from its solution. Chloroform was used as a solvent to blend two immiscible polymers, polycaprolactone (PCL) and polyurethane (PU), in a ratio of 70 to 30%, respectively. The solution was used in a high-air-pressure injection device which allowed the homogenization of the PCL and PU to form a microfiber structure. The resulting film was cut to form 1.5-cm × 3-cm laminates. To accomplish the “brick and mortar” architecture, each individual laminate (the “brick”) was then saturated with a water-dissolvable polymer (20% polyvinylpyrrolidone (PVP) solution in water for 1 min; the “mortar”), which acted as an adhesive to bond the polymer laminate layers. These polymer laminates were deposited one by one such that they formed a hollow cylindrical tube, with each individual laminate being saturated with the PVP solution then assembled over a cylindrical mold to form a resulting multilayered structure. After that, the mold was removed (by pulling it—which did not disturb the architecture of the resulting device) and the hollow cylindrical sample was left to dry for an additional 48 h. The completely dried device has a rigid structure due to the PVP polymer (Fig. 2). The device was designed and manufactured to have no inherent elasticity. The rigidity will allow the device to support the normal pressure inherent to the surgical procedures without bending or collapsing. During the drying process, the device was left with a relatively non-smooth and corrugated surface beneficial to the surgical process by preventing the soft tissues from excessively gliding over the surface. To the best of our knowledge, a device with a similar architecture and morphological structure has not been presented in the literature for use as a short-time implantation device.

#### Assessment of surface morphology and topography

A 3D Keyence Laser Microscope (LSCM, VK-X260K, Keyence, USA) was used to evaluate the surface morphology and topography of the guide. 3D measurement data were analyzed with Keyence’s Multi-File Analyzer software. Samples were examined in the following order: polymer laminate before saturation with PVP, polymer laminate after saturation with PVP. Both sets of samples were examined using 20X lens and 100X lens.

#### Mechanical testing

Compression tests of the samples were performed using the ADMET Expert 7601 universal testing system (ADMET, Inc., Norwood, Massachusetts). Briefly, the hollow cylindrical tube was placed horizontally in the sample holder. It was then subjected to a preload of 1 N before the compression test started, with a displacement rate of 10 mm/min and a maximum load applied of 1.1 kN. The sample was compressed up to about 100% of its initial diameter. After the data was collected, the stress/strain (displacement) curves were plotted.

#### In vitro device degradation or disassembly method

The device is intended to be a temporary supportive intraluminal anastomotic guide that can quickly degrade or disassemble within a desired timeframe—not less than 30 min and not more than 3 h after implantation in the intestine. To test the device’s ability to disassemble, we immersed the fabricated samples in a water bath and visualized device integrality with time.

### Swine model

Use of pigs for this study was carried out in accordance with all relevant regulations and guidelines, including those of ARRIVE, and was approved by the Institutional Animal Care and Use Committee at the University of Tennessee, Knoxville (#2522). 12 mixed-breed, white production pigs weighing between 14 and 40 kg (median 30.9 kg, mean 28.6 ± 7.2 kg) were utilized in this study, with standard anesthesia and surgical techniques. Pigs were randomly assigned to one of two treatment groups, and both study populations were similar with two exceptions. One pig was assigned to the control group because it was small in size and weight, which caused concerns that the bowel diameter would be insufficient to accommodate the pre-made, single-sized anastomotic guides. A second pig assigned to the control group had diarrhea post-shipping, which significantly improved after surgery. Each pig was fasted for a minimum of 12 h prior to surgery, and water access was restricted a minimum of 2 h before surgery. Peri-operative analgesia was provided by placement of transdermal fentanyl patches (1 µg/kg) along the dorsal midline in the mid-thoracic region at least 12 h prior to surgery. Subjects were pre-medicated with xylazine (2 mg/kg, IM), induced with a combination of midazolam (0.1–0.2 mg/kg, IM) and ketamine (10 mg/kg, IM), an endotracheal tube was placed, and anesthesia maintained using isoflurane (range 1 to 5%) vaporized into oxygen (100%). Each subject was placed into dorsal recumbency, clipped, and aseptically prepared along the ventral midline.

#### Control group

A complete, transverse enterotomy was performed across the jejunum perpendicular to the mesenteric border. Single interrupted stay sutures (#3-0 PDS, Ethicon, INC. Somerville, New Jersey), without knotting, were placed on the mesenteric and anti-mesenteric edges in order to hold the cut edges apposed. Anastomoses performed without a guide were initiated using two single, full-thickness simple interrupted sutures of #3-0 PDS, one placed on each of the mesenteric and anti-mesenteric borders. Then, a single row of full-thickness simple continuous sutures of #3-0 PDS were placed coursing from the anti-mesenteric margin to the mesenteric margin, followed by, in like manner, a single row on the opposing side from the mesenteric margin to the anti-mesenteric margin.

#### AG group

**Figure 3:**
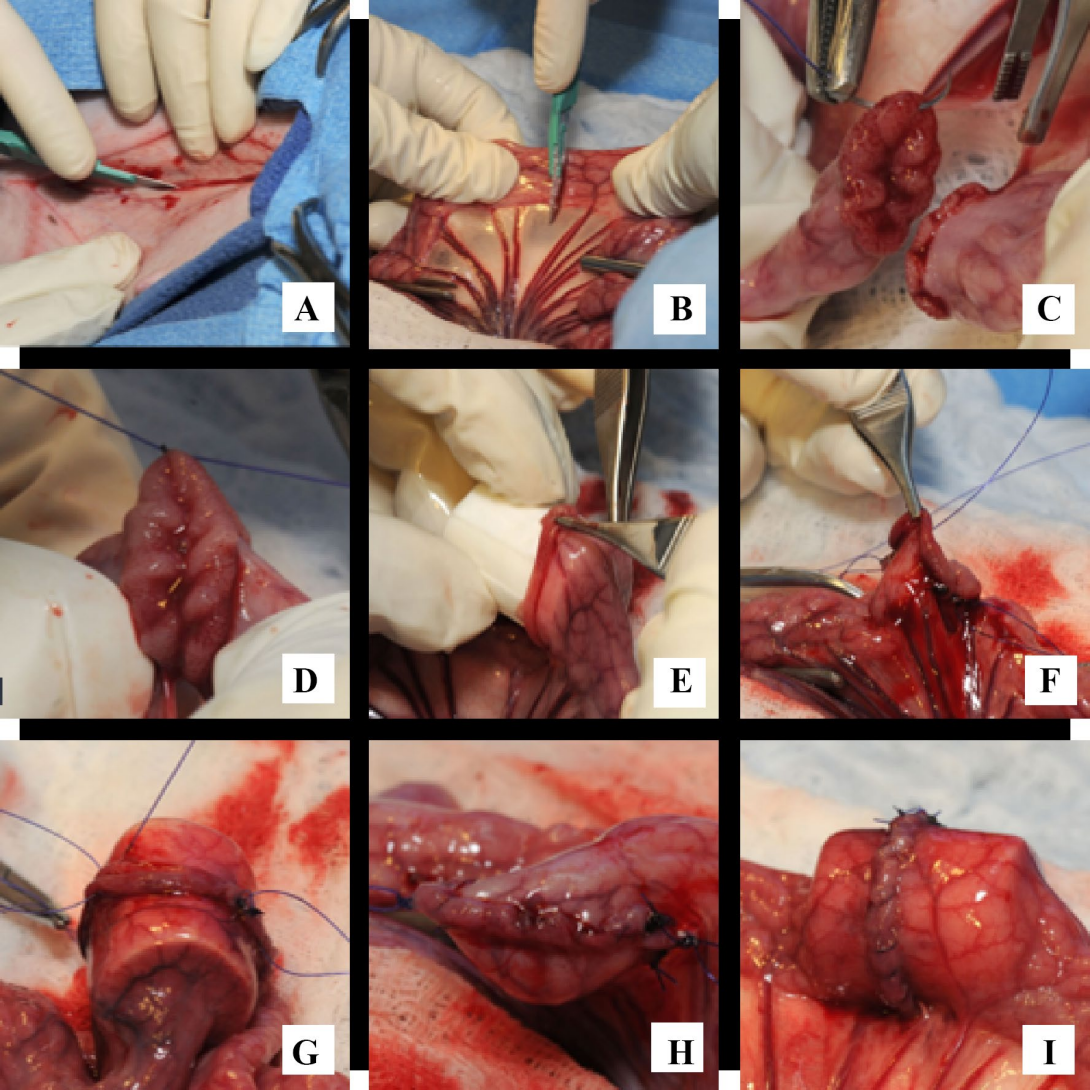
Serial images of surgical process; images pertaining independently to control procedure (“control”) or guide-facilitated procedure (“AG”) are indicated; (**A**) ventral midline incision; (**B**) enterotomy of isolated small intestinal section; (**C**) placement of interrupted suture on mesenteric side (control); (**D**) placement of interrupted suture on anti-mesenteric side (control); (**E**) placement of AG into intestinal lumen (AG); (**F**) completion of anastomosis in absence of AG (control); (**G**) completion of anastomosis overtop AG (AG); (**H**) completed anastomosis in absence of AG (control); (**I**) completed anastomosis with AG in situ (AG). Photo Courtesy of Phil Snow, University of Tennessee, College of Veterinary Medicine. All copyrights retained by University of Tennessee.

As depicted in Fig. 3, for anastomoses facilitated with an AG, the guide was placed into the lumen on one side of the enterotomy and then into the opposing side so that the bowel edges were opposed overtop of the guide. The suturing process was identical to that of the non-guide-aided anastomoses. Occasionally, an additional simple interrupted suture was placed between the mesenteric and anti-mesenteric edges to further secure the guide within the lumen. The integrity of each anastomosis was assessed by releasing the intestinal clamps and gently compressing contents from the surrounding bowel into the surgical site and monitoring for leakage. The bowel was rinsed with sterile saline and replaced into the abdomen, after which the linea alba was closed in a simple continuous pattern (#0 PDS, Ethicon, INC. Somerville, New Jersey). The skin and subcutaneous layers were closed together in a simple continuous pattern (#1 polypropylene, Ethicon, INC. Somerville, New Jersey). Total procedure time, starting with the initiation of the skin incision, and enterotomy time, starting with the first cut into the intestine, were recorded for all surgeries.

#### In vivo data

Fecal output was monitored with a scoring system twice daily for the duration of the study to analyze trends in consistency and track elimination of the anastomotic guides. The scale, adapted from Wen et al.^15^, ranged from 0 to 3, with (0) indicating normal/semi-firm feces, (1) pasty, (2) semi-liquid, and (3) liquid feces. If any pig were not to have defecated at the time of observation, no score was given.

#### Post-mortem data

Pigs were humanely euthanized 1 month after surgery, at which time intestinal burst pressure (Surgivet^®^ V6400 Invasive Blood Pressure Monitor, Smiths Medical PLC, Minneapolis, MN) and bowel diameter were measured and tissues collected for histologic assessment. Histology specimens were stained with hematoxylin and eosin (H&E) and Masson’s trichrome stain and evaluated by a veterinary pathologist. Assessed characteristics included deposition of collagen, inflammatory cell infiltration, width of the anastomotic site, and thickening of the serosa at the anastomotic site.

#### Statistical analysis

All quantitative analyses (total procedure time, total enterotomy time, return to fecal production, anastomotic site diameter compared to surrounding bowel, and burst pressure) were analyzed using a one-tailed student’s t-test, with *p*-value < 0.05 considered statistically significant. Averages are presented with standard deviations. Incidence of fecal score type post-surgery is presented as a percent of all fecal production recordings.

## Results

The devices were prepared as described in the experimental section and were then thoroughly characterized before being tested in the animal studies.

### Surface morphology and topography

**Figure 4:**
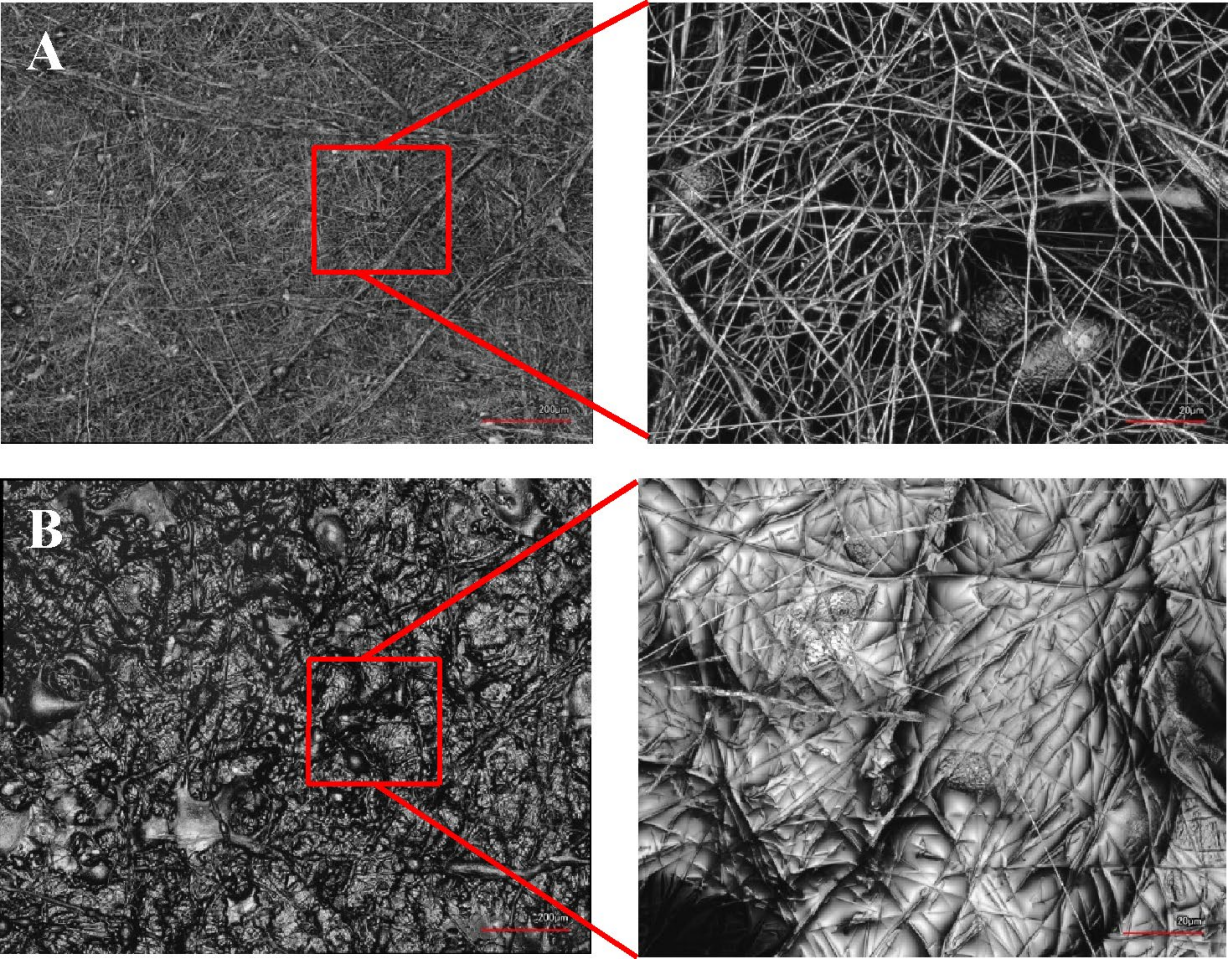
Representative 3D laser microscopy images: (**A**) the polymer laminate used to fabricate the device before saturation with PVP; the inset clearly shows the fiber structure; (**B**) the polymer film used to fabricate the device after saturation with PVP; the inset clearly shows the fiber structure where the laminate is partially coated with PVP polymer.

Surface morphology and topography of samples were evaluated using 3D laser microscopy. Two sets of samples were evaluated—the polymer laminate alone and the polymer laminate after saturation with PVP. The results confirmed that the polymer laminate alone had a porous structure with a fiber-like morphology (bundles), as shown in Fig. 4. The results also indicated a fiber structure for the polymer laminate used to fabricate the device after its saturation with PVP. Additionally, the PVP-coated polymer laminate demonstrated a fiber structure, which was clearly coated with the PVP polymer (Fig. 4B). There is a clear observation of the penetration of PVP polymer within the fibrous structure of the laminates, which allows the PVP to act as a “mortar” and hold the laminate “bricks” into place one on top of each other.

### Mechanical compression

**Figure 5:**
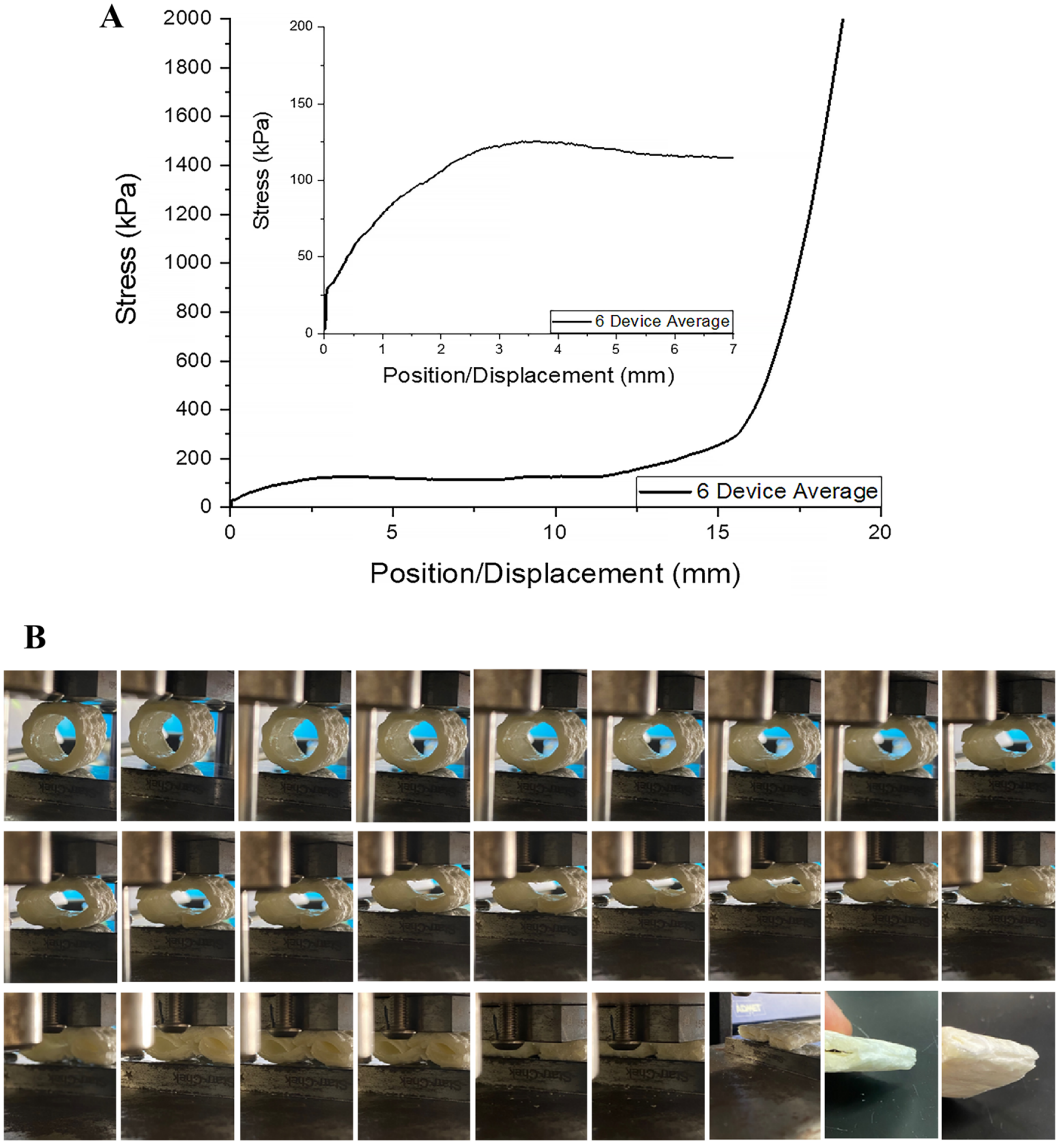
Mechanical compression results: (**A**) strain/displacement curve (average of 6 independent measurements)—the inset is the magnified part of the averaged curve for up to 7 mm displacement; (**B**) consecutive images collected during the compression of a device along with the final compressed image of the same device.

Compression testing was used to investigate the device’s durability and ability to maintain its structure during manipulation. It should be mentioned that the device was designed to be rather rigid, with little to no inherent elasticity. An elastic device, as it would bend under mechanical compression associated with the surgical process, would make it rather difficult for the surgeon to suture the tissues in the desired manner. As a result, the fabricated device is rigid before it collapses and disassembles once exposed to body fluids. The results indicated that the devices can withstand on average about 78 kPa when compressed 1 mm (4%) of the initial diameter, around 122 kPa when compressed to 3 mm (12%) of the initial diameter, and about 747 kPa when compressed by 17 mm (68%) of the initial diameter (Fig. 5).

#### In vitro device degradation or disassembly

**Figure 6:**
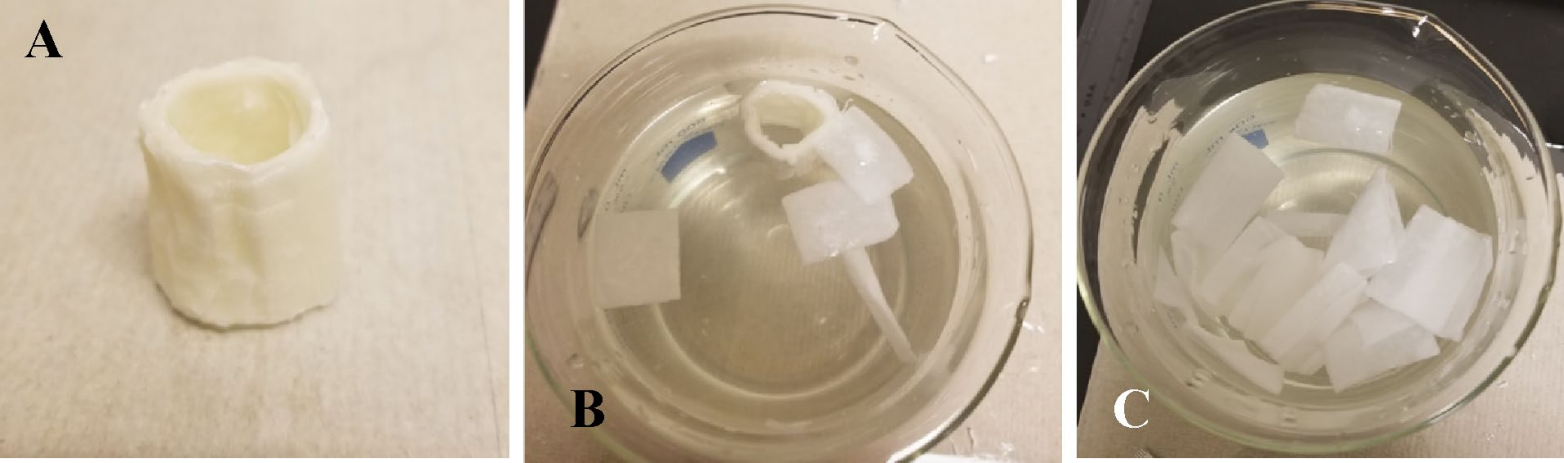
(**A**) Images of a fabricated device prior to placement in water bath; (**B**) the device starting to collapse/disintegrate after about 10 min in water; (**C**) the device totally collapsed after about 30 min.

The degradability or disassembly of the device when it comes into contact with water was evaluated using a water bath. The results showed that the device loses its integrity gradually after exposure to water. The disassembly starts when the water attaches to the adhesive polymer, which softens its structure, followed by separation of the outer layer of the device after 10 min. The device completely collapses or disassembles after about 30 min (Fig. 6) (Supporting Information, Video 1–3).

### In vivo application of anastomotic guide

#### Intra-operative data

##### Procedure time

The average procedure time for the control anastomoses was 47.2 min (± 5.3 min), compared to 48.8 min (± 5.8 min) for AG-facilitated anastomoses (p = 0.33). Enterotomy time for control anastomoses was 17.4 min (± 3.4 min) compared with AG anastomoses, which required a mean of 24.7 min (± 4.4 min). This difference was statistically significant (*p* < 0.05).

##### Surgeon’s observation

Subjective data from surgeons revealed an initial delay in adapting to utilizing the AG because of its novelty, but once the AG was placed within the lumen and the initial interrupted sutures completed, surgeons rated the performance of the anastomosis as enhanced compared with that of controls. The AG allowed for improved ease of placement of sutures and increased visualization of mucosal and serosal edges.

##### Fecal scoring

**Figure 7:**
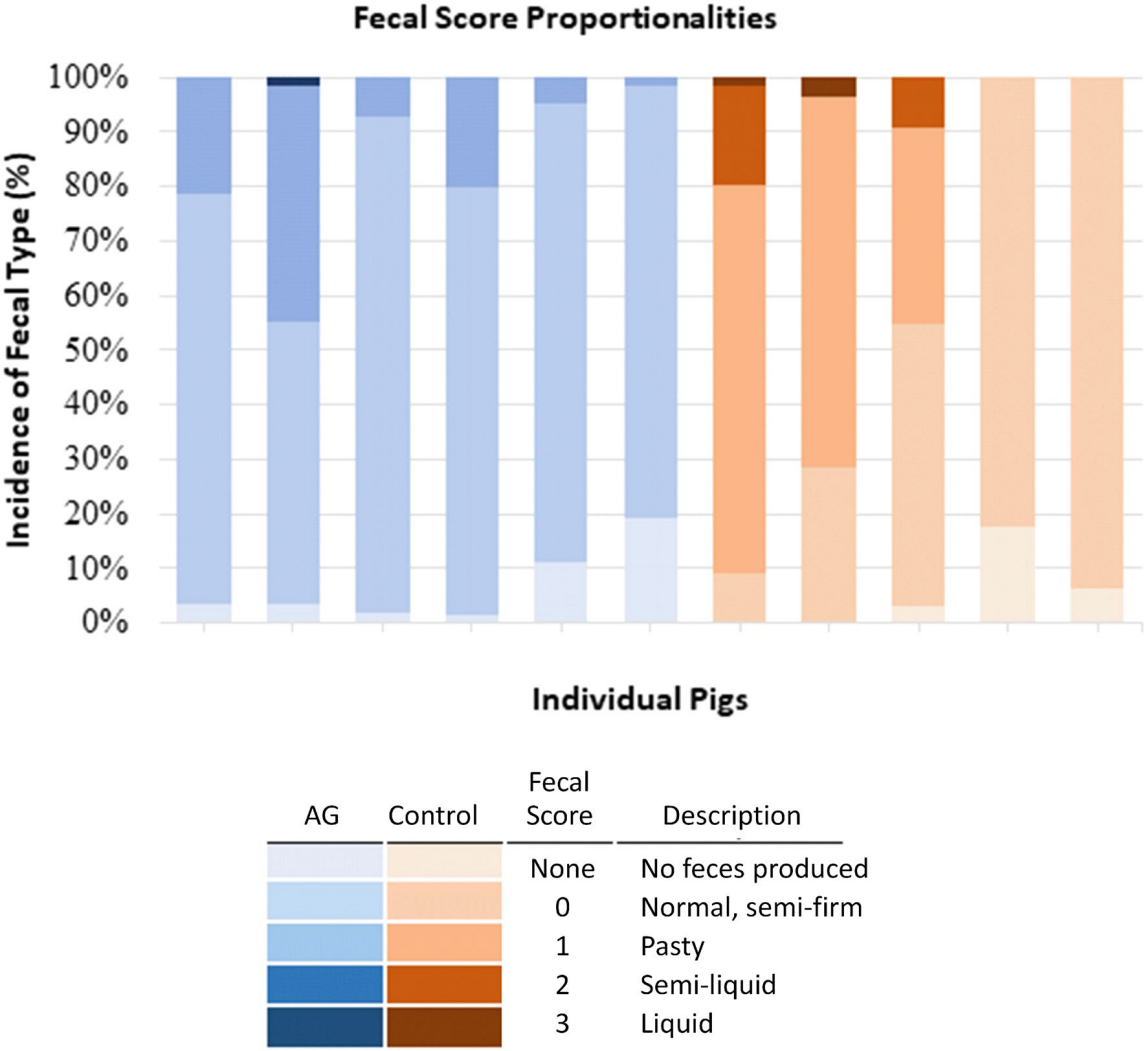
Incidence of fecal quality type within individual pigs based on fecal scoring performed at least twice daily. Incidence is represented as a percentage and refers to the number of fecal scores recorded within each category as compared to the total number of observations. Fecal score scale derived from Wen et al.^15^.

**Figure 8:**
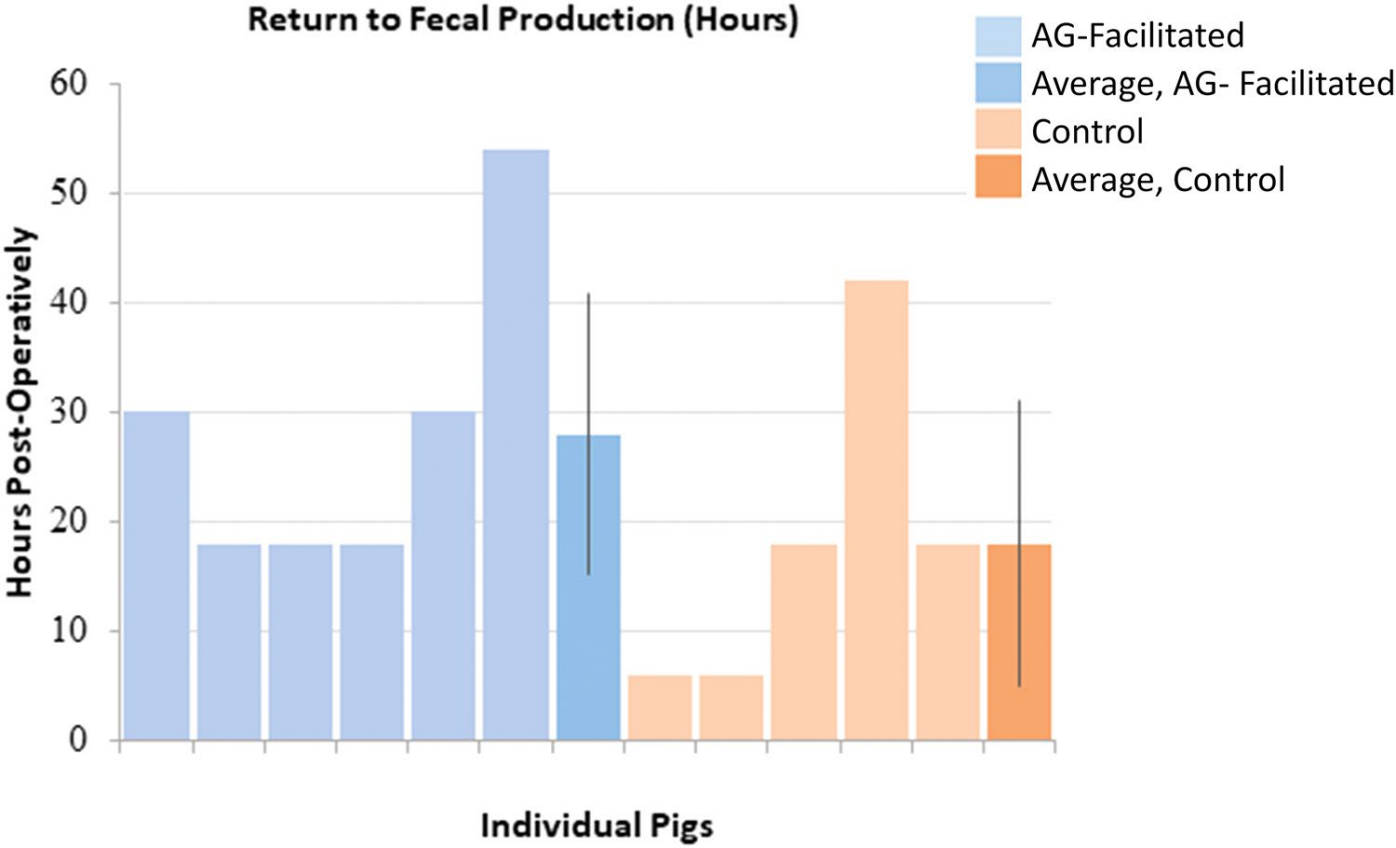
Hours post-operatively that individual pigs returned to fecal production. Black bars represent standard deviations of the averages for each group.

Fecal scoring (Fig. 7) and time to first fecal elimination (Fig. 8) revealed that fecal output returned by the second day after surgery in all pigs. On some occasions, feces exhibited different scores at different times on the same day. The highest fecal score was recorded for these samples and used for analysis. All pigs returned to fecal production at similar times and with similar fecal consistency. Of the pigs that received an AG, three passed the AG within 30 h of the procedure, one between 31 and 42 h, one between 43 and 54 h, and one between 90 and 102 h. All AGs were eliminated as individual, disbanded sheets. One of the pigs that had evidence of AG elimination within 30 h of the procedure passed additional remnants between 43 and 54 h.

##### Postmortem data

Occasionally, anastomoses were difficult to locate as a result of advanced incisional healing and minimal-to-no adhesion development. Seven pigs (3 control, 4 AG) had mild or moderate adhesions at the anastomotic site, and five pigs (2 control, 3 AG) had an adhesion elsewhere in the abdomen. Adhesions had no sign of impairing intestinal function and would likely not have resulted in motility disturbances in any of the pigs.

##### Intestinal diameter

**Figure 9:**
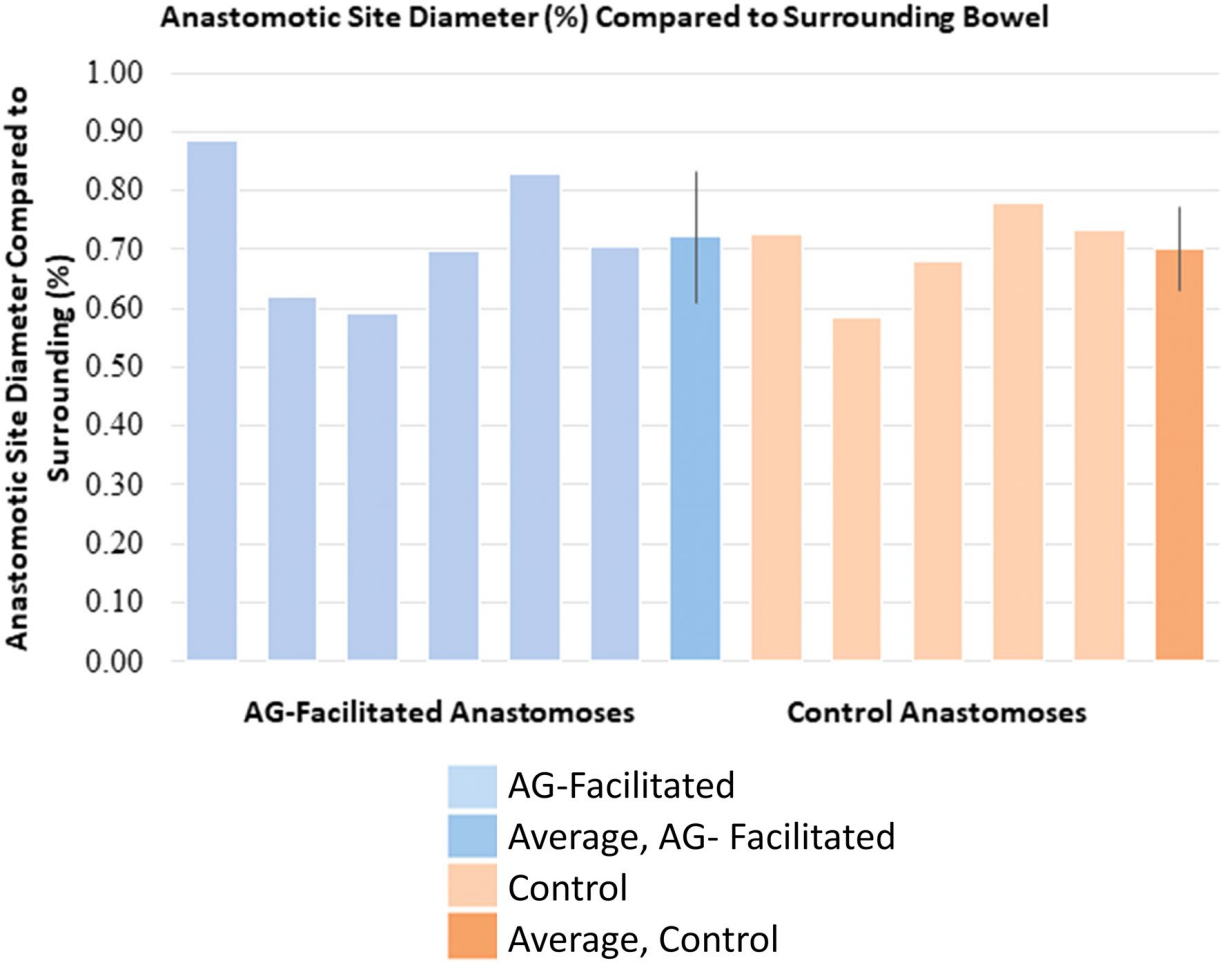
Diameter of anastomotic site in comparison to surrounding bowel for each pig. Percentage diameter reflects the diameter that the anastomotic site was when compared to the average diameter of the adjacent bowel (orad and aborad).

Segments of jejunum orad and aborad to the anastomotic site were clamped using Doyen forceps such that the anastomotic sites were located centrally. The segments were infused with saline until turgid, and the diameters of the anastomotic site, bowel diameter approximately 2-cm orad and 2-cm aborad to the end-to-end anastomosis (EEA) site, were measured with calipers. These values were considered the “maximum diameter” of the bowel in the respective regions. Within each sample, the average of the diameters of the orad and aborad segments was calculated and compared to the diameter of the anastomotic site, and the resulting percentage reflected the size of the anastomotic site in comparison to the surrounding bowel. A percentage less than 100 equivocates to relative stenosis at the anastomotic site, indicative of limited expandability from fibrous tissue or diameter reduction consequent of mucosal eversion during the anastomotic procedure itself. The range of anastomotic site diameter, as a percentage compared to the surrounding bowel, in the AG-facilitated group was 59 to 89% (mean, 72% ± 11%; Fig. 9). The range within the control group was 50 to 78% (mean, 70% ± 7%; Fig. 9). Anastomotic size was similar among treatment groups (*p* > 0.05). Additionally, percent difference between diameters of orad and aborad regions compared to diameters of anastomotic sites was determined, and the averages revealed statistically similar % difference on either side: 142% and 141% for the AG groups’ orad and aboard regions, respectively, and 149% and 139% for the control groups’ orad and aborad regions, respectively. These results clearly show that the AG did not induce any undesired or statistically relevant changes in the anastomotic site diameter or any differences in the orad and aborad regions compared to the controls.

##### Bursting pressure

**Figure 10:**
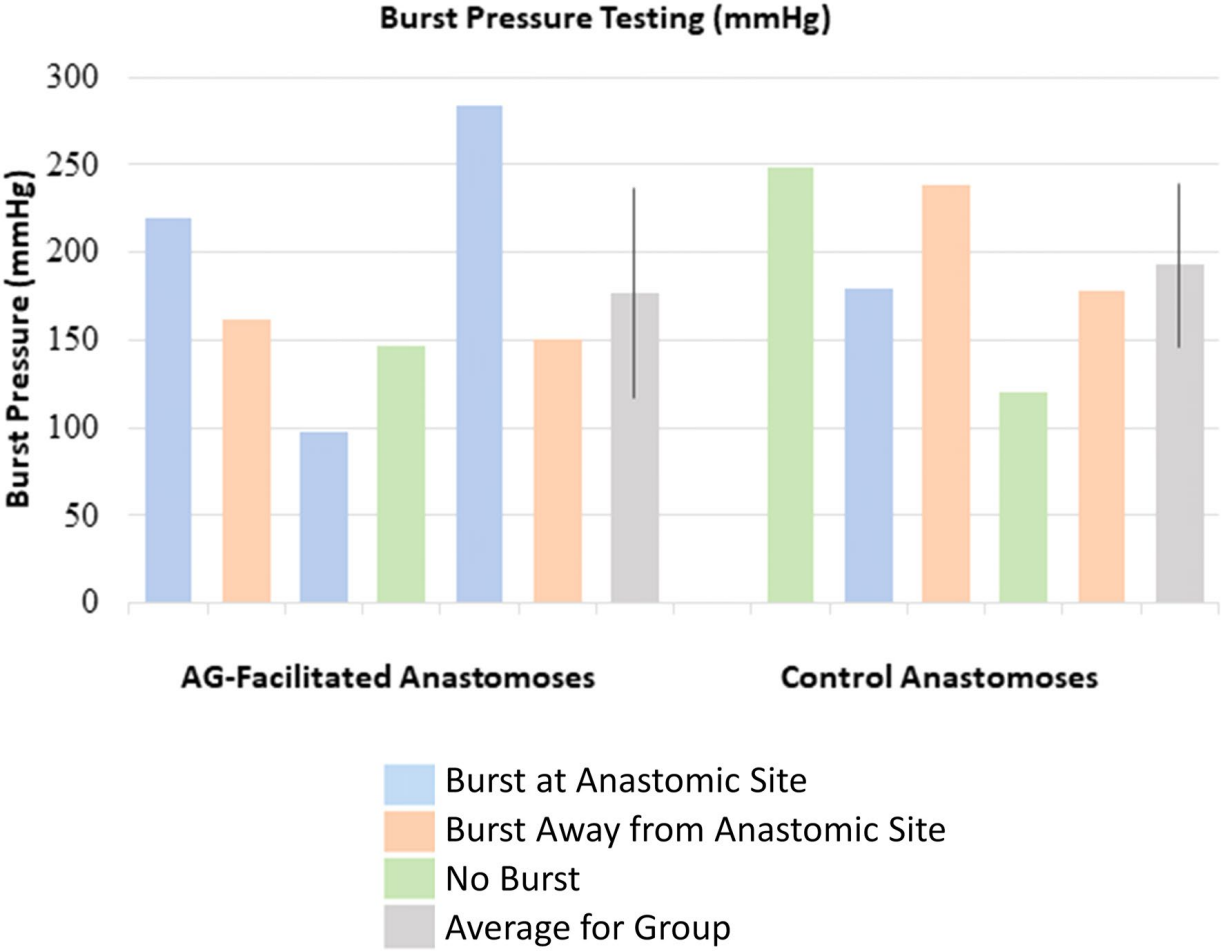
Burst pressures achieved by each anastomosis. Blue bars represent samples in which bursting occurred at the anastomotic site. Orange bars represent samples in which bursting occurred at the bowel adjacent to the anastomotic site but the anastomotic site remained intact. Green bars represent the maximum pressure achieved within the bowel lumen with no burst achieved. Grey bars signify the average for each group, and the overlying black lines represent standard deviations.

Intestinal wall burst pressure was obtained by infusing the segment with additional saline and monitoring the pressure within the lumen with a digital pressure monitor. Intestinal burst occurred at the anastomotic site in 4 out of 11 specimens (3 AG, 1 control). Bursting occurred in the bowel adjacent to the anastomotic site in 4 out of 11 specimens (2 AG, 2 control). Failure to reach burst pressure occurred in three specimens (1 AG, 2 control). Burst pressure was statistically similar for both treatment groups. The maximum value recorded was used as the maximum pressure for those specimens. AG burst pressures ranged from 97 to 284 mmHg (mean, 176.8 ± 59.9 mmHg), and control burst pressures ranged from 120 to 248 mmHg (mean, 192.8 mmHg ± 46.7 mmHg), as shown in Fig. 10. The values, however, did not show statistically relevant variations.

##### Histopathology

Slides were stained with H&E in order to demonstrate overall tissue architecture and cellularity. Histologic evaluation by a board-certified veterinary pathologist revealed minimal differences between samples overall and no profound abnormalities. The majority of samples displayed mild mucosal and/or submucosal mixed inflammation (eosinophils, lymphocytes, plasmacytes), at a level not unexpected in the porcine species. Most samples also revealed the presence of suture granulomas, distinguished by multilobulated macrophages, lymphocytes, and eosinophils congregated in an area where suture was previously present (or remained). Several samples, without a trend in group designation, also displayed mild lymphatic dilation. Two samples within the AG group had an area of hemorrhage, the cause of this being unknown, but at least one of which may have been due to manipulation of the tissue during harvesting and burst pressure testing. One sample from the control group was removed from evaluation due to improper sectioning.

## Discussion

Fabrication of the AG to the desired specifications was successful—initial sturdiness but rapid degradation once exposed to water or digesta. Device reproducibility was achieved through a novel fabrication approach in which layers of porous/fibrous polymer laminates were assembled to the form of a cylindrical shape over a diameter-controlled structure. This method also allows the device’s structural and morphological characteristics (such as size and shape) to be tuned based on user need. The expert surgeon opinion on the device is that it improved the ability to perform the hand-sewn EEA without significantly prolonging the total surgery time. The surgical benefits and usability of the device were described by one of the surgeons, Dr. Mulon, as follows: “The use of the intestinal guide helped tremendously to maintain alignment of the proximal and distal segments of the intestine, while concomitantly limiting the eversion of the jejunal mucosa. The sutures were performed very easily over the guide. Adding a temporary rigid intraluminal structure as a guide for end-to-end small intestinal anastomosis helped stabilize the intestinal ends and eased the realization of the sutures between the two intestinal segments”.

Use of the AG resulted in slightly longer enterotomy time, potentially associated with initial learning because of the device’s novelty. Despite this learning curve, once the AG was secured within the lumen, surgeons reported that performance of the anastomosis was enhanced. The AG allowed for enhanced visualization of the mucosa and serosa of the cut edges of the bowel, which may increase surgeon confidence that their suture was correctly placed. The ability of the AG to slightly dilate the bowel aids in the prevention of gaps within the repair, which can further increase surgeon confidence that post-operative leakage will not occur. The AG device not only enhanced surgeon ease, confidence, and visualization during the surgical procedures, but it also performed precisely as designed. All of the AGs disassembled (fell apart into individual components) after implantation and were passed out in feces by the animals in a reasonable time profile. One pig that experienced the longest delay in AG elimination also experienced the longest delay in return to fecal output, suggesting that return to normal intestinal motility was delayed. This is not uncommon after intestinal surgery. Post-mortem results showed no significant differences between the two groups in regard to adhesions, bowel diameter, intestinal burst pressure, or infection/leakage of the anastomosis. These findings clearly indicate that the AG did not induce any undesired effects in the animals during or after surgery.

The AG’s mechanical features were tuned for the needs of lumen expansion within the brevity of the procedure^6^. Although hydration tests were performed to assess disbanding of the sheets composing the AG, it was not possible to determine the disbanding rate in vivo. However, our ex vivo studies indicated that in a matter of 30 min or less, the AG devices disintegrated into the component polymeric sheets, which also showed a high degree of flexibility and no mechanical rigidity (Fig. 5). This data was collected with a non-sterilized guide that was fully saturated and exposed to frequent fluid flow dynamics to mimic the expected normal bowel movement in vivo. The lack of rigidity in the individual porous polymeric sheets was an intentional characteristic, designed to ensure effortless passage by the animals. Rigidity may have presented negative outcomes and caused internal damage with unforeseen results. The ultimate in vivo dismantling of the device is likely variable and dependent on the amount of digesta within the small intestine and the degree of peristalsis or presence of ileus within the bowel post-operatively. Another limitation is that since the guides are hand-fabricated and gas-sterilized prior to implantation, there could have been differences between their degradation characteristics.

Ultimately, this study demonstrates that this novel, rapidly degradable platform is functional in tissues with a lumen and in which the material can be passed out of the body. This platform serving as an AG can expand the lumen at the anastomotic site, enhance apposition of bowel edges, increase visibility of suture placement, and improve tissue handling without any added morbidities. This device may prove a beneficial addition to the surgical technique of small intestinal anastomosis.

## Supplementary Information

Supplementary Video 1.

Supplementary Video 2.

Supplementary Video 3.

## Data Availability

All data generated, collected, and presented as part of the current study will be made available by the corresponding authors upon reasonable request.

## References

[1] Middleton JC, Tipton AJ (2000). Synthetic biodegradable polymers as orthopedic devices. Biomaterials.

[2] Tobias KM, Ayres R (2006). Key gastrointestinal surgeries—Intestinal anastomosis. Vet. Med. Bonner Springs Then Edwardsville.

[3] Cha J (2015). Multispectral tissue characterization for intestinal anastomosis optimization. J. Biomed. Opt..

[4] Ashkanani F, Krukowski ZH (2002). Intestinal anastomosis. Surg. Infect. (Larchmt.).

[5] Chassin JL, Chassin JL (1994). Small bowel resection and anastomosis. Operative Strategy in General Surgery.

[6] Wang Z, Li N, Li R, Li Y, Ruan L (2014). Biodegradable intestinal stents: A review. Prog. Nat. Sci..

[7] Yao L (2016). An effective new anastomosis method. Med. Sci. Monit..

[8] Milan K (2018). The effect of three different surgical techniques for colon anastomosis on regional postoperative microperfusion: Laser Doppler flowmetry study in pigs. Clin. Hemorheol. Microcirc..

[9] Pedersen AP (2020). Development and in vivo assessment of a rapidly collapsible anastomotic guide for use in anastomosis of the small intestine: A pilot study using a swine model. Front. Surg..

[10] Hintz GC, Alshehri A, Bell CM, Butterworth SA (2018). Stapled versus hand-sewn pediatric intestinal anastomoses: A retrospective cohort study. J. Pediatr. Surg..

[11] Kano M, Hanari N, Gunji H, Hayano K, Hayashi H, Matsubara H (2017). Is, "functional end-to-end anastomosis" really functional? A review of the literature on stapled anastomosis using linear staplers. Surg. Today.

[12] Farrah JP (2013). Stapled versus hand-sewn anastomoses in emergency general surgery: A retrospective review of outcomes in a unique patient population. J. Trauma Acute Care Surg..

[13] Kuo WY (2017). Benefits of intraluminal agarose stents during end-to-end intestinal anastomosis in New Zealand white rabbits. Comp. Med..

[14] Williams DF (2009). On the nature of biomaterials. Biomaterials.

[15] Wen X (2018). Fecal scores and microbial metabolites in weaned piglets fed different protein sources and levels. Anim. Nutr..

